# Opportunities and challenges in interpretable deep learning for drug sensitivity prediction of cancer cells

**DOI:** 10.3389/fbinf.2022.1036963

**Published:** 2022-11-17

**Authors:** Bikash Ranjan Samal, Jens Uwe Loers, Vanessa Vermeirssen, Katleen De Preter

**Affiliations:** ^1^ Department of Biomolecular Medicine, Ghent University, Ghent, Belgium; ^2^ Center for Medical Genetics Ghent (CMGG), Ghent University, Ghent, Belgium; ^3^ Cancer Research Institute Ghent (CRIG), Ghent, Belgium; ^4^ Department of Biomedical Molecular Biology, Ghent University, Ghent, Belgium

**Keywords:** precision oncology, omics, deep learning, drug sensitivity prediction, interpretability, mechanistic insights

## Abstract

In precision oncology, therapy stratification is done based on the patients’ tumor molecular profile. Modeling and prediction of the drug response for a given tumor molecular type will further improve therapeutic decision-making for cancer patients. Indeed, deep learning methods hold great potential for drug sensitivity prediction, but a major problem is that these models are black box algorithms and do not clarify the mechanisms of action. This puts a limitation on their clinical implementation. To address this concern, many recent studies attempt to overcome these issues by developing interpretable deep learning methods that facilitate the understanding of the logic behind the drug response prediction. In this review, we discuss strengths and limitations of recent approaches, and suggest future directions that could guide further improvement of interpretable deep learning in drug sensitivity prediction in cancer research.

## Introduction

Cancer is a disease with multiple levels of complexity and the leading cause of mortality in EU countries after cardiovascular diseases (Nagai and Kim, 2017). First, for a given tumor entity, there is significant molecular variability across patients, which is referred to as inter-patient tumoral heterogeneity (Sanchez-Vega et al., 2018). Second, variability can also be observed within tumors, also termed as intra-tumoral heterogeneity (Marusyk et al., 2012). Third, under therapeutic pressure, tumors adapt through (epi-)genetic changes, which is defined as temporal heterogeneity (Venkatesan et al., 2017; Yu et al., 2021). With this knowledge in mind, therapy has gradually shifted from a one-size-fits-all approach towards precision oncology. The latter involves analysis of (epi)genome, transcriptome and proteome biomarkers that inform clinicians about the molecular characteristics of the tumor before and after therapy and aids in an improved diagnosis, prognosis, therapy stratification, and monitoring of the patient (Schwaederle et al., 2015). However, an important challenge in precision oncology is how to predict the drug sensitivity of a certain tumor based on its molecular make-up.

As it is ethically and practically unfeasible to compare the sensitivity of a panel of drugs on human cancer patient, different types of patient-derived cancer models such as cell lines, organoids, and patient-derived xenografts (PDX) are used instead. Cancer cell lines are the easiest to handle, and in general they also recapitulate the (epi)genetic and transcriptomic alterations as observed in the actual tumors (Kinker et al., 2020). These features make cell lines a widely used platform for drug screening (Tables 1 Tables 2 Tables 3). Large panels of cell lines that include a wide range of cancer types have been characterized at different omics levels (Barretina et al., 2012; Ghandi et al., 2019; Nusinow et al., 2020). Also, drug response profiles have been determined on these cell lines for a broad range of drugs (NCI60 (Shoemaker, 2006), CCLE (Barretina et al., 2012), GDSC (Garnett et al., 2012; Yang et al., 2012), CTRP (Rees et al., 2016), gCSI (Haverty et al., 2016)). These rich data resources can be harnessed to associate the phenotype (drug response) with the genotype of the tumors. Tables 1 Tables 2 Tables 3 provides a systematic view of the data resources available in pharmacogenomics that are supportive for predictive modeling of cell lines for drug sensitivity.

**TABLE 1:** An overview of drug sensitivity datasets. The table represents drug sensitivity datasets comprising of number of cell lines tested across panel of drugs, resulting in number of cell line–drug pair drug sensitivities, which is measured using a specific assay.

Dataset	Cell lines	Drugs	Drug responses	Assay
NCI60	60	52,671	3,780,148	Sulphorhodamine B
CCLE	479	24	11,670	CellTitre Glo
GDSC I	970	403	333,292	Resazurin or Syto60
GDSC II	969	297	243,466	CellTitre Glo
CTRP I	242	185	50,531	CellTitre Glo
CTRP II	860	481	395,263	CellTitre Glo
gCSI	409	16	6,455	CellTiter Glo

**TABLE 2:** An overview of the major omics datasets available for the cancer cell lines. The tabulated omics dataset from different studies are available in the DepMap portal (DepMapThe Cancer Dependency Map).

Dataset	Cell lines	Number of features
Transcriptomics (RNAseq)	1406	53,971 genes
Mutation	1771	18,784 genes
Copy number variation	1766	25,368 genes
Methylation (RRBS)	843	20,192 TSS sites
		54,531 CpG clusters
Proteomics (RPPA)	899	214 proteins
Metabolomics	928	225 metabolites
CRISPR gene dependency	1086	17,386 genes
Global Chromatin Profiling	897	42 histone modifications

**TABLE 3:** An overview of drug datasets.

Dataset	Data available
STITCH (Kuhn et al., 2007)	Drug-protein interaction information, chemical structure (SMILES and InChIKey)
Drug Bank (Wishart et al., 2006)	Drug target information, chemical structure (SDF)
PubChem (Kim et al., 2021)	Molecular properties, chemical structure (SDF, SMILES, InChi, InChiKey)
HMS LINCS KINOMEscan (HMS LINCS KINOMEscan dataa)	Drug binding strength across ∼440 kinases

**FIGURE 1:**
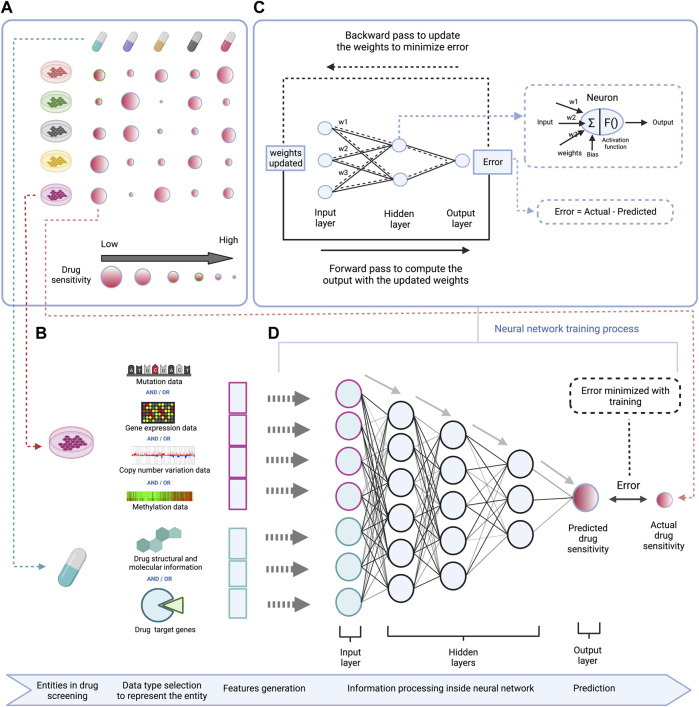
Schematic representation of a DL based model to predict drug sensitivity using omics features of cell lines and structural and molecular properties of drugs. **(A)** (left top)—High-throughput drug screening of cell lines across different drugs provides data for modeling drug sensitivity. **(B)** (left bottom)—Gene mutation, RNA expression, DNA copy number variation, and/or DNA methylation profiles for a given panel of genes are used as features to represent cell lines, and structural information like SMILES or Hashed Morgan fingerprint, molecular properties like molecular weight, solubility, lipophilicity, *etc.* or drug target information (e.g. among a set of genes, the drug targets vs. non-targets are represented in a binary form) are used as features to represent drugs in the DL model. **(C)** (right top)—Workflow demonstrating the training process of the DNN which involves adjustment of connection weights between the neurons to minimize the prediction error. **(D)** (right bottom)—A deep neural network is trained using the cell lines and drug features to predict drug sensitivity.

To train computational prediction models for drug sensitivity, both omics and drug sensitivity data of cell lines are used. The trained models can afterwards be applied on omics data of human tumors to predict drug vulnerabilities for individual patients (Lee et al., 2007). Various computational strategies are used to develop drug sensitivity prediction models, mainly machine learning based methods which include matrix factorization, support vector machines, random forests, and deep learning (Dong et al., 2015; Kim et al., 2019; Lind and Anderson, 2019). These methods generally use molecular information (omics data for a certain panel of genes) of the cell lines with structural and molecular information of the drug as input features, and the drug sensitivity as output/label for training (illustrated in Figure 1 for deep learning) (Menden et al., 2013).

Deep learning (DL) is a subset of machine learning that is based on artificial neural networks (ANN) (Goodfellow et al., 2016). The name and structure of ANN are inspired by the brain, simulating the way the biological neural network system works to process stimuli. An ANN is composed of an input layer, one or more hidden layers, and an output layer. When the ANN is composed of many hidden layers, it is referred to as a Deep Neural Network (DNN). One of the advantages of DNNs is that they can handle high-dimensional and noisy data. Furthermore, they learn input-output relationships incrementally through their hidden layers by transforming low-level features (raw data) to high-level features (embeddings), capturing nonlinear and complex relationships, which then are useful for output prediction. These capabilities give them superior predictive performance compared to many conventional machine learning algorithms. However, in most DL approaches, the data transformation inside the neural network is very complex and lacks interpretability. Therefore, the DL models are black box models, where the logic behind the predictions is hidden.

In this article, we review the recently published interpretable deep learning methods for drug sensitivity prediction that can be applied in precision oncology. We present how the interpretability of DL models could be an added value in rendering biological insights. We briefly introduce interpretability techniques and also describe and comment on interpretable DL methods for drug sensitivity prediction which include DrugCell (Kuenzi et al., 2020), HiDRA (Jin and Nam, 2021), PathDNN (Deng et al., 2020), PaccMann (Manica et al., 2019), consDeepSignaling (Zhang et al., 2021), DEERS (Koras et al., 2021), ParsVNN (Huang et al., 2021), DNN (Sakellaropoulos et al., 2019) and SWnet (Zuo et al., 2021). We address the factors that account for both strengths and limitations of these methods and discuss suggestions for further improvement.

## Interpretable deep learning

Despite the superior predictive performance of DL models, the lack of interpretability is an important shortcoming compared to other machine learning models. The model interpretability can be an essential added value. Firstly, it can explain how the model processes the input data to make the prediction. In addition, it can indicate if the model is paying proper attention to crucial input features which potentially influence the output prediction. Lastly, in case of prediction errors, it can explain why and how a model has malfunctioned and point to biases or artifacts present in the input data (Explainable, 2019). These interpretability features, in case of a drug sensitivity prediction model, can provide mechanistic insights to explain the drug mechanism of action (MOA) and the confidence in the prediction system. Hence, it will help the clinician with risk management for designing clinical trials, based on consistency between their expert knowledge and the model interpretability.

In recent years, many efforts have been made to develop strategies and techniques to interpret black box DL models. Three commonly used strategies are probing, perturbation, and surrogation (Azodi et al., 2020). The **probing** of a model is meant to discern the logic that the model has learned during the training**. The perturbing** strategy involves removing an input feature to see the effect on the output. The **surrogating** strategy is based on using an inherently interpretable model (e.g. a linear model) to approximate a black box model. Based on the intrinsic properties of a model, the interpretation can be performed at different levels, i.e. global, semi-global and local, and at different stages i.e. ad hoc and post hoc (see Table 4 for definitions).

**TABLE 4:** Glossary of terms related to the interpretation of machine/deep learning models.


Global interpretation	Interpretation explaining predictions of the whole dataset
	Example: All cell lines vs. all drugs
Semi-global interpretation	Interpretation explaining predictions of a subset of the dataset
	Example: all cell lines vs. one drug, or *vice versa*
Local interpretation	Interpretation explaining prediction of an instance of the dataset
	Example: a cell line—drug pair combination
Ad hoc interpretation	Interpretation at the time of prediction due to the model’s inherent ability
	Example: Using neural embeddings that emerged during drug sensitivity prediction to identify the activity of genes and pathways to explain the drug mechanism of action (HiDRA (Jin and Nam, 2021))
Post hoc interpretation	Interpretation that is based on statistical tests performed on the prediction results or the learned model parameters
	Example: Gene set enrichment analysis on learned model weights to identify the pathways involved in MOA for a given drug (DNN (Sakellaropoulos et al., 2019))

**FIGURE 2:**
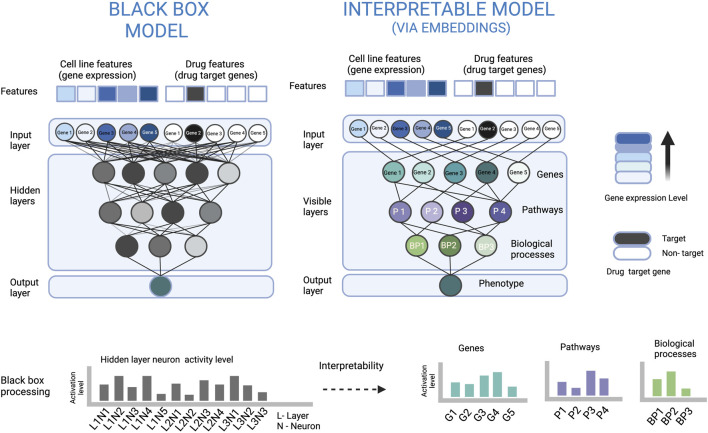
An illustration to show how embeddings at neurons of a biologically constrained ANN can be used for interpretation. In the left part, the densely connected ANN (black box model) is very complex, which makes it difficult to understand how the input is processed to arrive at the output. In the right part, the sparsely connected ANN based on some biological hierarchy of genes, pathways and biological processes makes the data processing visible and also explains the relevance of each neuron and its output. For example, the node of Gene 3 has a high activation level, and the signal only from the Gene 3 node is passed to the Pathway 2 node. The latter has a low activation level which can be interpreted that the pathway is inactive, and Gene 3 has a suppressing effect on Pathway 2.

Nearly all the interpretable DL based drug sensitivity prediction studies available are based on the probing strategy. Since it is easier to describe the interpretability strategy using example methods, we only focus on the probing strategy in this review. The reason why probing is widely applied is that it can explain how the input data is processed inside the neural network, what input features got more attention to predict a specific output, and that it can justify the relevance of transformed data at each node/layer of the neural network (explained in Figure 2). There are mainly three techniques used in the probing strategy i.e. embeddings, weights, and gradients, which we will address in detail in the next section.

## Three classes of probing based interpretable deep learning

### Embeddings

The embedding at a neuron can be regarded as the representation of inputs of the neuron in the form of its output. Therefore, the embedding at neuron can be represented by the neuron output. The neuron embedding sometimes, is also referred to as the neuron state or the activation level (Azodi et al., 2020; Deng et al., 2020; Kuenzi et al., 2020; Lin and Lichtarge, 2021). The output of the neurons can be used as a means to interpret the model and this is useful when the complete or a part of the ANN architecture is based on some constrained connections between the neurons e.g. some form of biological organization where a neuron node or neurons in a layer represent an actual pathway or biological process (illustrated in Figure 2).

#### DrugCell

Kuenzi et al. developed Drugcell for predicting drug sensitivity in the context of cancer by adopting principles of the previously published method DCell which predicts yeast cell growth from its gene deletion genotype (Ma et al., 2018). The DrugCell model is developed to fulfil two objectives. The first objective is to predict drug response for a given drug on a given cell line using mutational profiles of the cell line and the chemical structure of the drug as features. The second objective is to use the trained model to explain the drug MOA, suggesting important pathways implicated in the drug response. The DrugCell model architecture consists of two ANNs, one for the genotype and another for the drug structure that are combined to predict the drug sensitivity. The ANN for the drug structure is made up of fully connected layers (i.e. not constrained). The ANN for the genotype is structured in such a way that it mirrors the hierarchy of biological processes inside a cell, by constraining the connections inside the ANN using the Gene Ontology hierarchical information. The constrained connections help to represent the hidden layers as actual biological processes and render them biologically interpretable.

**FIGURE 3:**
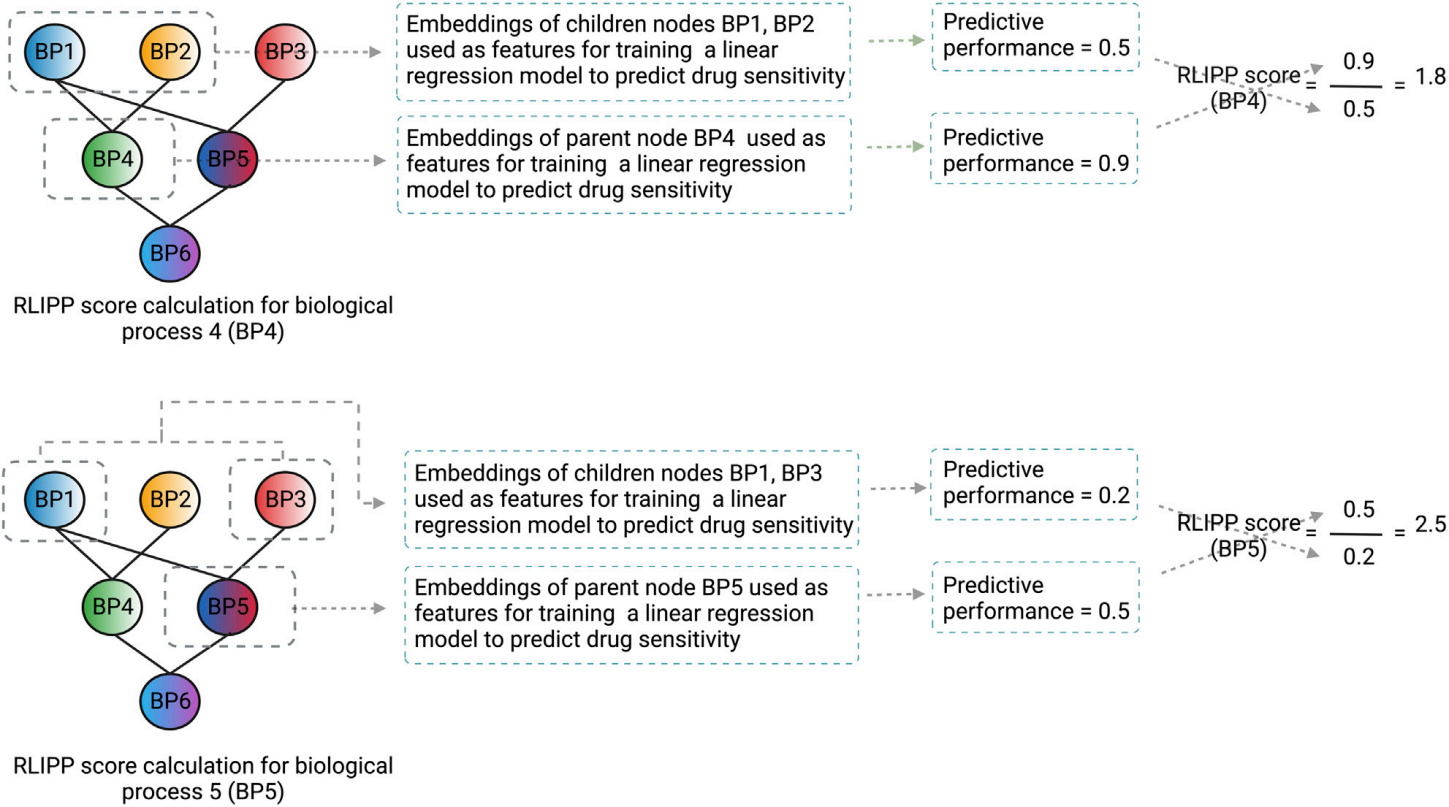
An illustration of how the RLIPP score can be non-informative in a given setting. The GO biological processes BP4 and BP5 are at the same hierarchical level and their respective RLIPP scores are computed. Although the predictive power of BP4 is greater than BP5, BP5 has a greater RLIPP score than BP4. Therefore, a high RLIPP score for a biological process does not guarantee that it will be more informative and important for the prediction, rather it will only say how much the biological process has more or less predictive power than its children processes. The color scheme of the nodes in the neural network represents the flow of signals from the children biological processes to the parent biological process and the color fusion symbolizes the fusion of signals from the children to the parent node. Each node here represents a layer in the genotype ANN and the name, and the structure of the layers are based on GO hierarchy of biological processes.

After the model is trained on the whole dataset, a post hoc interpretability analysis is performed by computing the Relative Local Improvement in Predictive Power (RLIPP) score for each of the GO biological process term layers in the genotype ANN. The RLIPP scoring system gives a semi-global interpretation, in which it ranks a set of GO terms as important signatures for a specific drug across all cell lines (cancer types). However, this RLIPP scoring may not be a good way to rank GO biological processes in all cases because scores of biological processes may not be comparable among each other in order to identify the top processes (illustrated in Figure 3).

The authors validated the model interpretability using external RNA-seq data of the 25 most sensitive *versus* the 25 most resistant cell lines against the drug docetaxel. For this, they performed a differential gene expression analysis and GO Biological Process enrichment using DAVID (Huang et al., 2009a; Huang et al., 2009b). The obtained list of overrepresented pathways was then compared with the list of pathways obtained from the model post hoc analysis and notably, they were found to be distinct. However, the authors claimed that the experimental validation was convincing. As the model suggested “Response to cAMP” as a top pathway, they treated A427 cells with three different treatments–paclitaxel, the glycolysis inhibitor 2-deoxyglucose, or a combination of the two, and found that the combination was substantially more effective than either individual compound.

#### HiDRA

Jin et al. introduced a DL model called Hierarchical network for Drug Response prediction with Attention (HiDRA) to predict drug sensitivity of cancer cell lines (Jin and Nam, 2021). In the ANN architecture, they employed a hierarchical attention network that showed highly attended biological pathways and their member genes related to the drug response. The hierarchical attention here means while passing input data from gene-level to pathway-level and then from pathway-level to output in the neural network, the attention mechanism figures out which part of the data is more important than others at the respective levels for the prediction. The model consists of four ANNs: a drug network, a gene-level network, a pathway-level network, and a response prediction network. The gene-level network and pathway-level network have an attention module for calculating the importance of genes and pathways, respectively. The model uses gene expression profiles as features to represent the cell lines and the drug structure in the form of hashed Morgan fingerprint as features to represent the drugs.

HiDRA suggests a set of pathways and the pathway member genes that are important behind a specific cell line-drug response prediction: hence this approach performs interpretation at the local level. This is realized by looking at the attention scores of pathways and their member genes derived from the pathway-level network and the gene-level network, respectively.

The validation of the interpretability was done by performing a case study with Gefitinib and Rapamycin on the LB2241-RCC cell line. The log fold-change of the genes’ attention score was computed for Gefitinib in comparison to Rapamycin. It was found that the target genes of Gefitinib had high positive log fold-change, whereas the target genes of Rapamycin had high negative log fold-change, inferring that the respective target genes received more attention and are important for the respective predictions. The case study only showed a gene-level analysis, skipping pathway level analysis. Moreover, the attention scores were not directly used (contrary to the claim), rather their relative change between two drugs was used to identify the important genes behind the drug sensitivity prediction.

#### PathDNN

Deng et al. developed a Pathway-guided Deep Neural Network (PathDNN) to predict the drug sensitivity in cancer cell lines (Deng et al., 2020). The ANN architecture is structured by constraining the connections between the gene layer (input layer) and the pathway layer (first hidden layer) based on gene-pathways relations obtained from the KEGG pathway database. The model input layer is divided into two parts. The first part takes gene expression data of a certain set of genes, representing the cell line features. The second part takes binary data of a certain set of genes indicating whether they are drug targets or not, to represent drug features. The drug targets were retrieved from the STITCH database (Kuhn et al., 2007).

The model interpretation is based on post hoc analysis of the neuron output of the pathway nodes in the pathway layer. A comparison is made between the output of the pathway nodes with drug treatment and those without drug treatment and quantified in terms of log2 fold-change. The no-drug treatment setting is made by setting drug features equal to zero. For a given cell line–drug pair, the log_2_ fold-change is computed for all pathway nodes and the pathways with the highest log_2_ fold-change are considered as important pathways responsible for drug response prediction. The model is locally interpretable as it can explain a single cell line–drug pair.

The authors validated the interpretability performance of their method with a case study of eight rhabdomyosarcoma cell lines treated with the CTK7H7014 drug. One important remark is that the specified drug cannot be found back in any drug database. Moreover, no gene target information is available for the drug and therefore the mode of action is not known. In the case study, the log_2_ fold-change (drug vs. no-drug) of the neuron output of 323 pathways was computed for each of the eight cell lines. This analysis revealed that the Hsa05202 pathway, which is related to rhabdomyosarcoma, frequently occurred among the top pathways across the eight cell lines that were treated with the drug. However, the other top pathways were not further discussed in the study.

#### PaccMann

Manica et al. adopted their previous work on the Prediction of AntiCancer Compound sensitivity with Multimodal Attention-based Neural Networks (PaccMann) and modified it with a novel architecture that uses attention based multiscale convolution encoders (Manica et al., 2019). The model uses two encoders, a gene expression encoder and a SMILES encoder (Weininger, 1988). The input to the gene expression encoder is gene expression profiles of a set of most informative genes. These genes are selected using a network propagation scheme carried out on the STRING PPI network. For each drug, the weights initialized (=1) to the reported drug targets are diffused over the STRING network, resulting in an importance distribution over the genes. The important genes obtained for each of the drugs are merged to form a set of most informative genes. The SMILES encoder takes SMILES embeddings plus the output embeddings from the gene expression encoder as input. The output from both the encoders are further connected to a feed-forward layer to predict drug sensitivity. As a result of using the output of the gene expression encoder into the SMILES encoder, for a given cell line across all the drugs, the gene attention values remain constant and the attention values over SMILES encoding change.

The model interpretability is based on a post hoc analysis of gene attention values from the gene expression encoder. The genes that are frequently attended across all cell lines are used for pathway enrichment analysis to investigate which pathways are induced by the drugs. The analysis identified that the apoptosis signaling pathway is elicited in general by all anti-cancer drugs present in the dataset. Since this interpretation assumes that every drug has the same drug MOA across all cell lines, it gives a global interpretation.

The authors presented a case study to validate the method based on the sensitivity of the MEG-01 cell line for Imatinib and Masitinib. They showed how the model predicted differently between two different drugs on the same cell line. Based on attention values over the SMILES encoding, they determined what molecular substructures of the drug were important for the sensitivity prediction. They also aimed to show what genes were important for the prediction for a given cell line-drug pair. However, this may be misleading because for a given cell line the model will show the same attended genes across all drugs, so it will not be possible to distinguish drug-specific MOA among different drugs.

### Gradient

**FIGURE 4:**
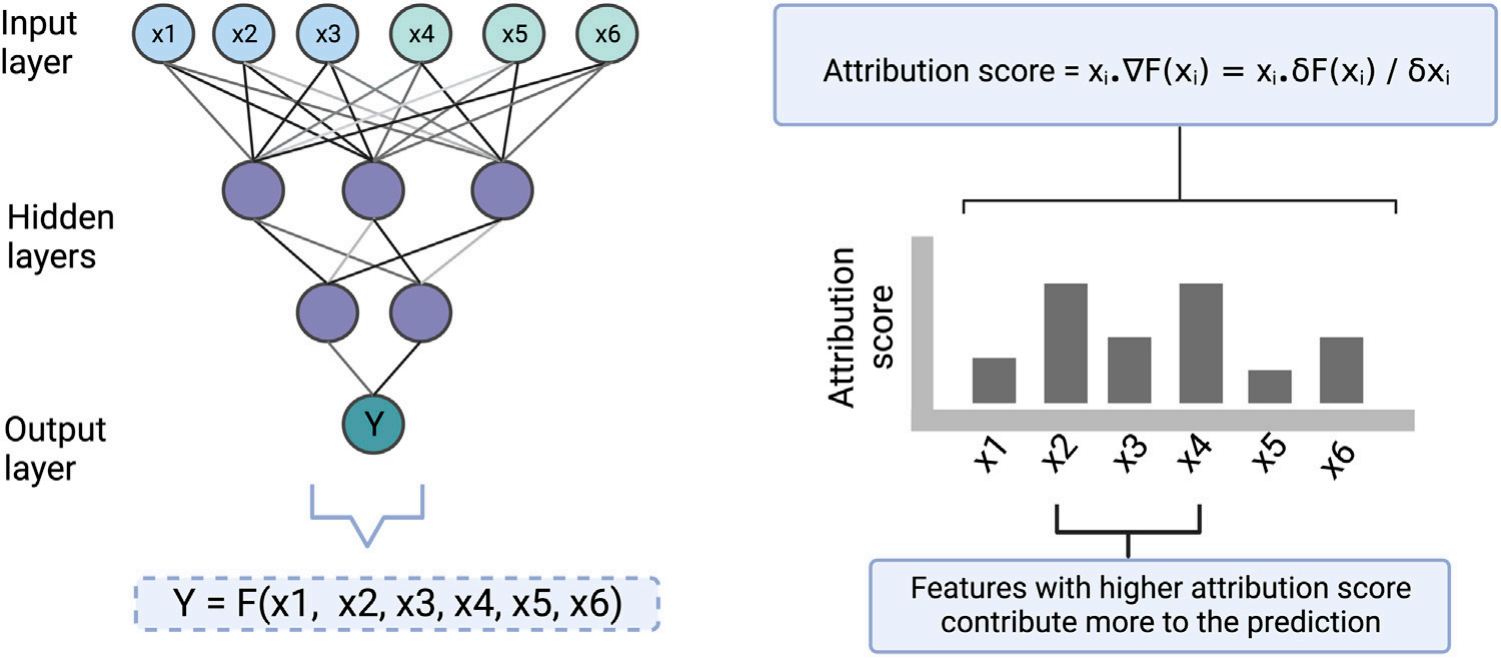
An illustration to show how the gradient-based approach scores the input features which are then used for making the interpretation. Here [x1, x2, x3] and [x4, x5, x6] represent cell line and drug features respectively. Y represents drug sensitivity. F represents a function in the form of a neural network that takes [x1, x2, x3, x4, x5, x6] as input and returns Y as an output. The gradient of the model with respect to each of the input features is computed to find the attribution of the features towards the output. ∇F(xi) represents the gradient of the function at xi which is also equal to the partial derivative of the function with respect to xi. The computed attribution score for a feature tells us how much the feature contributes to a prediction.

The feature importance score based on gradient is determined by calculating the change in the predicted output upon a small change in an input feature, using partial derivatives (illustrated in Figure 4). The gradient-based approach has the limitation that it is not useful when small changes in the feature value have no effect on the output prediction. Moreover, individual features may sometimes not have any effect on the output but may have an effect when combined. Therefore, this approach only explains the individual feature relationship to the output, which is a second limitation. However, this technique is applicable to all kinds of ANN architecture and hence it is a flexible approach.

#### consDeepSignaling

Zhang et al. developed a constrained neural network architecture guided by signaling pathways, called consDeepSignaling for performing drug response prediction on cancer cell lines (Zhang et al., 2021). The ANN is structured by constraining the connections between the input layer and the pathway layer (first hidden layer) based on gene-pathways relations obtained for all available 46 signaling pathways from the KEGG pathway database. The model uses a set of genes related to the pathways as features, where each gene is represented by its gene expression, copy number variation, and drug target-gene binary call.

The post hoc interpretability analysis of the model is based on investigating the importance of signaling pathways behind the drug response prediction using the SmoothGrad model in the “iNNvestigate” python package (Alber et al., 2018). This tool is used to extract the gradients of the pathway layer in the trained model to score the importance for each of the pathways. The model interpretability does not explain the drug MOA but rather reported what signaling pathways are important across all predictions, thus providing a global interpretation.

The publication of Zhang et al. did not attempt to include validation for the interpretability performance of the model.

#### DEERS

Koras et al. developed a neural network recommender system for kinase inhibitor sensitivity prediction, called Drug Efficacy Estimation Recommender System (DEERS) (Koras et al., 2021). The ANN architecture contains two autoencoders to represent cell line and drug features into low dimensional representation and a feed forward ANN to combine them for drug response prediction. The model uses gene expression, binary mutation calls, and tissue type information as features to represent the cell lines, and binding strength of the drug across 294 protein kinases obtained from HMS LINCS KINOMEscan data resource as features to represent the drugs (HMS LINCS KINOMEscan data). This method can be considered a hybrid of both gradient and embeddings based interpretation.

The post hoc analysis for interpretation is done at the feature and the biological process level. At the feature level, for a given drug, the input features’ attribution towards the final output are computed for each cell line separately using the Integrated Gradients method (Sundararajan et al., 2017). The attribution scores are then averaged across all cell lines. The averaged attribution gives a semi-global interpretation as it cannot explain at a specific cell line level, but instead explains for all cell lines together for a given drug. At the pathway level, each dimension of the low dimensional representation from the cell line autoencoder is assigned to biological processes by correlating it with the expression of each gene across all cell lines, followed by GSEA on the ranked correlation values. The same dimensions are also correlated with the sensitivity for a given drug across all cell lines, to associate drug response with biological processes.

The authors used a case study with three drugs (PHA-793887, XMD14-99, and Dabrafenib) to validate the interpretability of the method. They computed the averaged feature attribution to show the top cell line and drug features important for the prediction for a given drug. They observed that the drug target gene was present among the top drug features for all three drugs, but the top cell line features did not include relevant genes (cancer or tissue type related gene), except for Dabrafenib. They also showed which biological processes best represented the drug sensitivity across all cell lines for a given drug, by performing a correlation analysis between the predicted drug sensitivity and hidden dimensions embedding. Almost the same biological processes (hidden dimensions) are associated with the drug sensitivity for the three drugs. However, the authors did not explain how these biological processes are related to the drug sensitivities for each of the drugs based on literature evidence.

### Weights

**FIGURE 5:**
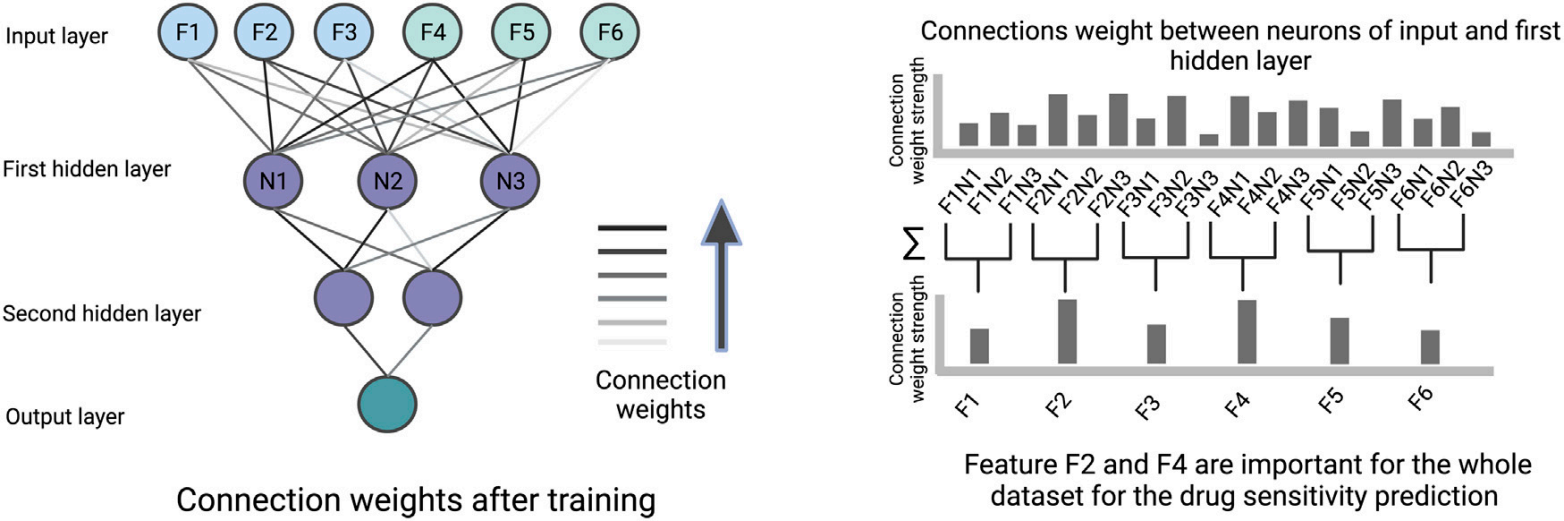
An illustration of how connection weights between the neurons in an ANN can be used for interpretation. In this figure, the neuron weights between the input layer and the first hidden layer are used to identify important features determining the prediction.

The connection weights between an input layer neuron, representing a specific cell line or drug feature, and the neurons of the first hidden layer can be used to quantify the importance of this feature by summing over the learned weights between them (illustrated in Figure 5). Therefore, the first hidden layer is interpretable based on weights and the remaining part of the ANN has no role in the interpretability. The features with high weights could be interpreted as the more important components for the prediction. The feature importance scores based on weights can be misleading when features are on different scales, when positive and negative connection weights cancel each other out, or when a connection has a large weight but is rarely activated.

#### ParsVNN

Huang et al. introduced parsimony visible neural network (ParsVNN) for cancer type specific drug sensitivity prediction and interpretation (Huang et al., 2021). The authors believed that the biological hierarchy used in DrugCell is agnostic to the downstream prediction task, as some of the functional components in the biological hierarchy are not involved in the biological process related to the drug sensitivity phenotype. The conventional learning algorithm used in DrugCell also does not distinguish the redundant and irrelevant functional components which may permit them to make contribution towards the prediction. Therefore, such redundant and uninformative functional components in the visible neural network architecture can lead to overfitting and also result in misleading interpretations. To address this problem, the authors have built cancer-type specific model by pruning the redundant and irrelevant components for that cancer-type. To do this, they introduced sparse inducing penalty terms to the loss function and employed proximal alternative linearized minimization (PALM) algorithm as an optimization method to minimize the loss function and learn the model parameters. The penalty terms help to prune the components with less important weights. The cancer-type specific model is achieved by training the model with the cancer-type specific training data. The architecture and features used in ParsVNN are exactly the same as in DrugCell, which includes the same genes and subsystems (biological process GO terms) to build the visible neural network.

The interpretability for a given cancer-type specific model is based on post-hoc analysis to identify the non-zero connection weights between the components. This reveals the components i.e. the gene nodes and the subsystem nodes that remain in the parsimonious architecture which contribute to the drug sensitivity prediction. Since this approach explains the predictions for a given cancer entity across a panel of drugs, it gives a semi-global interpretation.

The authors validated the interpretability for five different cancer types (stomach, breast, pancreatic, kidney and liver cancer) at both gene level and subsystem level. The genes and subsystems nodes remained in a cancer-type specific parsimonious model were hypothesized to be the driver genes and the prognostic biological processes for that cancer type, respectively. The authors validated the first part of the hypothesis by checking the degree of overlap between the identified genes and the cancer-specific driver genes reported by IntOGen pipeline (Martínez-Jiménez et al., 2020). The second part was validated by analyzing each of the leaf subsystems (GO terms) in cBioPortal’s survival analysis with the cancer-type specific samples. The samples were divided into two groups, where one group had the samples that did not contain any gene mutated among the member genes of the GO term, and the other contained at least one gene mutated among the member genes of the GO term. The authors found that some of the GO terms that had its member gene(s) mutated significantly influence the clinical outcome.

#### DNN

Sakellaropoulos et al. developed a conventional deep neural network model (DNN) using gene expression data for a panel of highly variable genes as features to predict drug response (Sakellaropoulos et al., 2019). For each drug, the model training is performed on cancer cell lines, and the prediction is done on cancer patients.

The interpretability based on post hoc analysis of the model assigns biological meaning to the nodes of the first hidden layer. The weights are extracted from the nodes of the first hidden layer that are connected to gene nodes in the input layer. The weights are used to perform gene set enrichment analysis and the normalized enrichment score of every node is calculated against every pathway in the Reactome database. The normalized enrichment score for each significant pathway across the nodes of the first hidden layer is plotted as a heatmap. The nodes are clustered into subgroups, where each subgroup shows its signature of enriched pathways, suggesting possible drug mechanisms. The training and interpretability analysis is done for each drug separately. The interpretation is qualitative because the drug signature pathways are inferred from cluster patterns. The authors claimed that the pathways inferred from the analysis for cisplatin, paclitaxel, and bortezomib, are consistent with the literature evidence.

#### SWnet

Zuo et al. developed a DL predictive model called Self-attention gene Weight layer Network (Swnet), which uses gene expression, gene mutation, and drug structure data to predict drug sensitivity (Zuo et al., 2021). The model consists of a gene branch and a drug branch. The drug branch uses a graph neural network to convert the 2D representations of chemicals into embeddings in the latent space. The gene branch uses a gene weight layer in the form of a weight matrix to integrate the information of gene expression and gene mutation. The weight matrix is based on self-attention formed on the chemical similarity between all drugs, and this accounts for the heterogeneity of the gene-drug relationship. The integrated information is then processed using a convolution neural network leading to transformation into embeddings in the latent space. The embeddings from the drug and genomic branches are integrated and processed by another CNN to transform it into a unified output.

The model interpretation is based on post hoc analysis of the gene weight layer, where the genes with a specific *weight* = 1 are identified for each drug. The authors expected that the proteins encoded by these genes would interact with the drug targets and consequently a protein-protein interaction database can be used to validate the existence of the interaction.

The authors performed a validation of the approach with case studies on BRAF and BCL2 inhibitors. The genes identified in these case studies (BRAF and BCLS) were shown to be only two edges away from the drug target gene in the PPI network, which was interpreted as a validation by the authors. As shown in supplementary data, most of the genes identified for all other drugs were found not to be related to the drugs or the targets.

## Discussion

While DL methods have proven their value in precision oncology applications (Coudray et al., 2018; Campanella et al., 2019; Chen et al., 2020), lack of interpretation of these black box models makes it difficult to implement them in clinical practice. In the past years, research has shifted focus toward interpretable DL and several methods have been developed to predict drug sensitivity. Table 5 gives an overview of the different published approaches. The methods cover different techniques for interpretation of the models, and each of them has certain advantages and limitations that we will discuss in this section. However, it is important to mention that it is not possible to precisely compare the methods based on their predictive performance, as they use different metrics and cross validation schemes for performance evaluation.

**TABLE 5:** Summary of studies describing interpretable deep learning approaches for drug sensitivity prediction. It includes the used datasets and types, the validation approach, the model drug sensitivity prediction performance, the model interpretability parameters and the programing language and packages used to develop the model. DrugCell, PathDNN and PaccMann have online web portal which makes their usability easier for a clinician with no knowledge of programming. Abbreviations: GMBCP, Gene mutation binary call profile; GEP, Gene expression profile; CNVP, Copy number variation profile; HMFP, Hashed Morgan Fingerprints; DTBC–Drug Target Binary Call; DBS, Drug binding strength; CV, cross-validation; LOCOV, Leave one cell line out validation; PCC, Pearson correlation; R2, Coefficient of determination; Rho, Spearman correlation; MSE, Mean squared error.

Sl. No.	Model	Data type	Dataset	Validation split	Independent set validation	Performance	Neural network type	Interpretability technique	Type of interpretability	Programming language, software package(s) and/or online web portal
1	DrugCell	Cell line: GMBCP (3008 genes)	GDSC and CTRP	^a^5-fold CV	No	Rho = 0.80	Constrained	Embeddings	Semi-global Interpretation	Python
		Drug: HMFP	1235 cell lines and 684 drugs							PyTorch
		Drug sensitivity: Normalized AUC							Post hoc analysis	drugcell.ucsd.edu
2	HiDRA	Cell line: GEP (4592 genes)	GDSC1000	5-fold CV	GDSC1	PCC = 0.9307	Constrained	Embeddings	Local Interpretation	Ad hoc analysis
		Drug: HMFP			GDSC2					Python
		Drug sensitivity: IC50	968 cell lines, 235 drugs		CCLE	R2 = 0.8647				Keras
3	PathDNN	Cell line: GEP (537 genes),	GDSC	10-fold CV	CCLE	R2 = 0.42	Constrained	Embeddings	Local Interpretation	Python
		Drug: DTBC (741 genes)	970 cell lines, 250 drugs	LOCOV		PCC = 0.798			Post hoc analysis	PyTorch
		Drug sensitivity: Normalized AUC								pathdnn.denglab.org
4	PaccMann	Cell line: GEP (2128 genes)	GDSC	25-fold CV	No	Test performance on lenient splitting	Unconstrained	Embeddings	Global Interpretation	Python
		Drug: SMILES	985 cell lines, 208 drugs			PCC = 0.9284			Ad hoc analysis	Tensorflow
		Drug sensitivity: IC50	Lenient split with 5-fold CV			R2 = 0.8619				ibm.biz/paccmann-aas
5	consDeepSignaling	Cell line: GEP, CNVP (929 genes), Drug: DTBC (929 genes)	GDSC	5-fold CV	No	PCC = 0.85	Constrained	Gradient	Global Interpretation	Python
		Drug sensitivity: AUC	791 cell lines, 24 drugs						Post hoc analysis	Keras, iNNvestigate
6	DEERS	Cell line: GEP (202 genes), GMBCP (21 genes), tissue type	GDSC	^a^Train- ∼42,000 instances	CCLE	Using IC50, PCC = 0.82	Unconstrained	Gradient	Semi-global Interpretation	Python
		Drug sensitivity: DBS (across 294 protein kinases)	922 cell lines and 74 drugs	Test and Validation - ∼5000 instances		Using AUC			Post hoc analysis	PyTorch
		Drug Sensitivity: Normalized IC50, and AUC				PCC = 0.76				Captum
7	ParsVNN	Cell line: GMBCP (3008 genes)	GDSC and CTRP	Train:Test- 80:20%	No	NA	Constrained	Weights	Semi-global Interpretation	Python
		Drug: HMFP	205 cell lines and 684 drugs	5-fold CV					Post hoc analysis	Pytorch
		Drug sensitivity: Normalized AUC								
8	DNN	Cell line: GEP, Drug Sensitivity: IC50	GDSC	5-fold CV	Clinical data	NA	Unconstrained	Weights	Semi-global Interpretation, Post hoc analysis	R, H_2_O
			1001 cell lines, 251 drugs							
9	SWnet	Cell line: GEP (1478 genes)	GDSC	Train:Test- 90:10%, LOCOV	No	GDSC	Unconstrained	Weights	Semi-global Interpretation, Post hoc analysis	Python, PyTorch
		GMBCP (1478 genes)	983 cell lines, 221 drugs			MSE = 0.9384				
		Drug: Molecular graph	CCLE			R2 = 0.8683				
		Drug Sensitivity: IC50	469 cell lines, 24 drugs			CCLE				
						MSE = 1.1604, R2= 0.7283				

^a^ Drugcell and DEERS are trained on the full dataset before the interpretability step.

The comparison of the different methods indicates that the important ingredients of the interpretable DL models for drug sensitivity prediction are the input data, the ANN architecture, and the ontological information on genes and pathways.

The quantity and quality of the training/testing datasets influence the predictive performance. Xia et al. showed that machine learning models trained on the CTRP dataset (887 cell lines and 544 drugs) showed more accurate predictions than models trained on GDSC (cell lines 1075 and 249 drugs) (Xia et al., 2022). First, GDSC shows more replicate variability in drug response assay than CTRP. Second, CTRP contains a larger number of drugs and cell lines, allowing the models to better capture a large diversity of relationships between cell processes and drugs. Moreover, multiple omics data are available for cell lines, and this can be beneficial in the learning and improving the model performance (Malik et al., 2021). However, not all omics and all drug sensitivity assays are characterized for all cell lines, which could be a slight limitation for not having muti-omics training data as large as single-omics training data.

The ANN architecture determines whether the interpretation is done in a post hoc or ad hoc manner and what resolution of interpretation can be achieved. For example, HiDRA has the ad hoc ability to render interpretation at the time of prediction, at both pathway and gene-level resolution. The design of an ANN architecture also depends on the objective of the study. For example in PaccMann, the architecture is designed to explain which drug substructure is responsible for drug sensitivity, whereas in other methods the architecture is designed to explain what pathways or genes are responsible for drug sensitivity.

The gene and pathway ontological information in the form of pathways gene set and pathways hierarchical relationships that are used to design the ANN determines the quantity of information i.e. number of features and the depth and the type of interpretability. For example, HiDRA uses the highest number of genes among all the methods, which could provide more information to the model for a deeper resolution of the interpretation. Another example is consDeepSignalling, where the authors wanted to explain the prediction in terms of signaling pathways, therefore they used signaling-pathway gene sets to design the ANN.

Among the three probing techniques discussed, the embeddings based approach is the most promising. It avoids the pitfalls of the gradient and the weight based approach. Moreover, the embeddings based approach can explain MOA at gene, pathway, and biological process level behind a drug response by allowing visible data processing inside the ANN. The gradient and the weight based technique can only reveal the important features behind a prediction. Moreover, the comparison of the discussed DL methods also shows what different levels of interpretation the three probing techniques can confer. The embeddings based technique can offer local, semi-global and global interpretation, using ad hoc and post hoc analysis. It is important to note that ad hoc analysis can only provide local interpretation. The gradient based technique can also offer interpretation at all three levels, but it can only be achieved through post hoc analysis. However, it can be applied on both constrained and fully connected ANN. The weight based approach can only offer global and semi-global interpretation using post hoc analysis.

There are many issues regarding the validity of the interpretability observed across the methods. Quantitative methods for testing the accuracy of the predicted biological interpretability are lacking. The interpretability remains mainly at a qualitative level, checked with anecdotal evidence from the literature. Moreover, genes or pathways in the top list that had an agreement with the literature are prioritized, and other genes or pathways are completely ignored. Another issue is that the majority of the methods offer a semi-global interpretation to explain the drug MOA, picturizing that all cancer types show the same signature pathways for a given drug. Similarly, the methods that had global interpretation showed that all cancer types across all drugs will have the same signature drug pathways.

The difficulty with the validation of interpretability is that there is no good source of ground truth available. The L1000 study can be considered a potential source for ground truth, where it provides the drug signature pathways at different drug concentrations and time points for a given cell line (Subramanian et al., 2017). In the case of DL methods, binarized labels or IC50/AUC values are used as a measure of drug sensitivity for training and prediction. These drug sensitivity metrics represent an overall effect of the drug, therefore the interpretability will also explain the overall MOA. Since the L1000 explains concentration and time point specific drug MOA, it complicates its use as ground truth.

In one study, interpretable DL was already successfully implemented in vitro studies, where its mechanistic insights are used to design and test drug combination therapy and drug repositioning (Kuenzi et al., 2020). As a proof of concept, DrugCell used its interpretability to discover a synergistic drug combination of Paclitaxel and 2-DG on A427 cells and discovered in *in vitro* studies that the combination was indeed more effective than either individual compound.

Although there are already many studies on interpretable DL in cancer research, there is still room for improvement. In precision oncology, patient-specific treatment is the ultimate goal; therefore, focusing on locally interpretable methods to predict drug sensitivity at a personalized level is very relevant. Moreover, to address drug resistance in the context of intra-tumoral heterogeneity, interpretable models at the single cell level would allow to gain ultraprecise mechanistic insights that could help in designing patient-specific drug combinations. Last but not least, it is noteworthy to mention that the developments of interpretable techniques in cancer research could also be useful in other diseases which we have not discussed in this review.
