# Supplementary material for: Opportunities and challenges in interpretable deep learning for drug sensitivity prediction of cancer cells
Source: Front Bioinform. 2022 Nov 17;2:1036963. doi: 10.3389/fbinf.2022.1036963 (PMC9714662; doi:10.3389/fbinf.2022.1036963)
Supplement: Supplementary file 1 [file DataSheet1.PDF]

Mathematical Frameworks:

1. DrugCell[1]

Neural Network Configuration:

Each subsystem in the hierarchy of subsystems in DrugCell, is assigned a number k of neurons to represent its multidimensional state.

$O^{(s)} = f(W^{(s)} . I^{(s)} + b^{(s)})$

where:
$O^{(s)}$ is subsystem state represented as a function of the states of its c child subsystems and *g* directly annotated genes.
$W^{(s)}$ is a weight matrix of dimensions and $k \times (k * c + g)$.
$b^{(s)}$ is a weight vector of dimension k.
$W^{(s)}$ and $b^{(s)}$ provide the parameters to be learned for subsystem s.
$\mathbf{I}^{(s)}$ is concatenation of of he states of c child subsystems and *g* directly annotated genes.
f is a non-linear transformation based on hyperbolic tangent and batch normalization.

Training of parameters is performed using:
Loss function = mean-squared error
Optimizer = SGD

2. HiDRA[2]

Neural Network Configuration:

The attention module of gene-level network for calculating the importance of each gene:

$$u_{ij} = \tanh(W_i C_i + b_i)_j$$

$$\alpha_{ij} = \frac{\exp(u_{ij}^{\mathrm{T}})}{\sum_j \exp(u_{ij}^{\mathrm{T}})}$$

$$p_i = \sum_j \alpha_{ij} g_{ij}$$

where:
C_i is a concatenation vector of G_i and D,
W_i is the weight of the attention layer in the gene-level network for pathway i, and,
b_i is the bias of the attention layer in the gene-level network for the pathway.

The attention module of pathway-level network for calculating the importance of each pathway:

$$u_i = \tanh(W_p Q + b_p)_i$$
$$\alpha_i = \frac{\exp(u_i^{\mathrm{T}})}{\sum_i \exp(u_i^{\mathrm{T}})}$$
$$q_i = \alpha_i \odot p_i$$

where:
Q is a concatenation vector of $P = \{p_1, p_2, ..., p_{186}\}$ and drug descriptor D,
W_p is a weight of the attention layer in the pathway-level network, and
b_p is the bias of the attention layer in the pathway-level network.

Training of parameters is performed using:
Loss function = mean-squared error
Optimizer = ADAM

3. PathDNN[3]

Neural Network Configuration:

Pathway layer = First hidden layer (323 neurons)

Connections between the input layer and the pathway layer = Associations between genes and pathways using 323*1278 mask matrix (M)

In the back propagation process, the weights are iteratively updated using the rule as below:

$$W_{\mathrm{mask}} = W_{\mathrm{feedback}} {}^{*} M$$

where:
W_{feedback} represents the feedback weights in the last backpropagation iteration, and
W_{mask} represents the successive forward weight which asserts the sparsity of gene-pathway association.

The feed-forward process is iteratively calculated as below:

$$a_{n+1} = f(W_{n+1}^{\mathrm{T}} a_n + b_{n+1})$$

where:
W_{n+1} is the weights between hidden layers H_n and H_{n+1}, b_{n+1} is the bias vector, and
f is the activation function that performs nonlinear transformations.

Training of parameters is performed using:
Loss function = mean-squared error
Optimizer = ADAM, SGD

4. PaccMann[4]

Network Propagation

The propagation function:

$$W_{t+1} = \alpha W_t A' + (1 - \alpha) W_0$$

where:
D is the degree matrix and
A' is the normalized adjacency matrix, obtained from the degree matrix *D*:

$$A' = D^{-1/2} A D^{-1/2}$$

Neural Network Configuration:

Self-Attention (SA)

The SMILES attention weights α_i were computed as

$$\alpha_i = \frac{\exp(u_i)}{\sum_j^T \exp(u_j)} \quad \text{where } u_i = V^T \tanh(W_e s_i + b)$$

Contextual-Attention (CA)

The contextual attention weights α_i are computed as :

$$u_i = V^T \tanh(W_e s_i + W_g G) \quad \text{where } W_g \in \mathbb{R}^{A \times |G|}$$

Training of parameters is performed using:
Loss function = mean-squared error
Optimizer = ADAM

5. consDeepSignaling[5]

Neural Network Configuration:

Input layer node vector :
$X= [x1,f1,x1,f2,\ldots x1,fK,\ldots,xi,fk\ldots,xn,fK] \quad T \in \mathbb{R}Kn\times 1$,
where:
K equals the number of features for each gene,
n equals the number of genes, and
xi, fk denotes the kth feature of ith gene.

Gene layer node vector:
$G=(g1,g2,\ldots,gi\ldots,gn)\in\mathbb{R}n\times 1$
where:
n equals the number of genes
gi denotes ith gene.

Pathway layer is denoted by $P=(p1,p2,\ldots,pi,\ldots,ps)\in\mathbb{R}s\times 1$,
where s equals the number of signaling pathways.

Feature connection matrix /mask matrix $CXG\in\mathbb{R}Kn\times n$:

$$C_{XG} = \begin{bmatrix} c_{1,1} & 0 & \cdots & 0 \\ \vdots & \vdots & \cdots & \vdots \\ c_{K,1} & 0 & & 0 \\ \vdots & \vdots & \ddots & \vdots \\ 0 & 0 & & c_{Kn-K+1,n} \\ \vdots & \vdots & \cdots & \vdots \\ 0 & 0 & & c_{Kn,n} \end{bmatrix}.$$

Information passing from Input layer to Gene layer:
$(CXG \cdot WXG)TX = G$

Information passing from Gene layer to Pathway layer:
$(CGP\cdot WGP)TG = P$

Training of parameters is performed using:
Loss function = mean-squared error
Optimizer = ADAM

6. DEERS[6]

Training of parameters is performed using:
Loss function = $J(WW)$

$J(WW)$ = MSE($\mathbf{y}-\hat{\mathbf{y}}$)$+rD\cdot$MSE($XXD-XX'D$)
$+rC\cdot$MSE($XXC-XX'C$)
$+d\cdot\sum m,n,m\neq n(KKD[m,n])2$

$+d \cdot \sum m,n,m \neq n(KKC[m,n])2$,J(WW)=MSE(y−y^)
+rD·MSE(XXD−XXD′)
+rC·MSE(XXC−XXC′)
+d·∑m,n,m≠n(KKD[m,n])2
+d·∑m,n,m≠n(KKC[m,n])2,

where :
J is the cost function,
MSE denotes mean squared error,
WW is a set of the model parameters (weights),
rD is the real-valued weight of the drugs reconstruction error,
XXD is the drugs' data matrix in the training batch,
$XX'D$ is the drugs data reconstruction matrix in the batch, rC is a real-valued weight of the cell lines reconstruction error,
XXC is the cell lines data matrix in the batch, $XX'C$ is the cell lines data reconstruction matrix in the batch,
d is a weight of the dependence penalty,
KKD is the covariance matrix of drugs hidden representations in the batch, and
KKC is the covariance matrix of cell lines hidden representations in the batch, and
$KK[m,n]$ denotes the *m*, *n*th entry of matrix KK.

Optimizer = ADAM

7. ParsVNN[7]

Neural Network Configuration:
Same as DrugCell

Training of parameters is performed using:
Loss function = L(WI,WS)+Ωλ(WI)+Γη(WS)
where:
$\Omega\ \lambda(W^I)$ and $\Gamma\ \eta(W^S)$ are sparse inducing penalty terms

Optimizer = Proximal Alternative Linearized Minimization (PALM)

8. DNN[8]

Neural Network Configuration:

f(α) = Output of a neuron
where:
f is a nonlinear activation function

$\alpha = \Sigma n_{i=1}\ w_i x_i + b$
where:

w = Connection weights
x = Inputs
b = bias

Training of parameters is performed using:
Loss function = mean-squared error
Optimizer = NA

9. SWnet[9]

Neural Network Configuration:

GNN (for molecular graph) edge transition:

$e_{ij}^{(t+1)} = \sigma(e_{ij}^{(t)} + g_{ij}^{(t)})$

$g_{ij}^{(t)} = f(w_e(v_i^{(t)} + v_j^{(t)} + b_e))$

where :
σ is the element-wise sigmoid function and
f is a non-linear activation function like ReLU, $we \in R^{d\times d}$ and $be \in R^{d}$ are the trainable parameters and bias vector respectively,
d is the dimension of edge embedding vector.

GNN (for molecular graph) node transition:

$v_i^{(t+1)} = \sigma(v_i^{(t)} + \sum j_{\in N(i)} h_{ij}^{(t)})$

$h_{ij}^{(t)} = f(w_n [v_j^{(t)} \;\; e_{ij}^{(t)}] + b_n)$

where:
$wn \in R^{d\times 2d}$ and $bn \in R^{d}$ are the trainable parameter matrix and bias vector respectively,
N(*i*) is the set of neighboring indices of *i*.

The arithmetic mean of all node embedding from transition function :

$h_G = 1/N \sum^{N}_{i=1} v_i^{(t)}$

where *N* is the number of nodes in the graph

Weight matrix to evaluate the significance of genetic mutation:

$geneCom = geneExp + geneVar * w'$

where:
geneCom is the combination of expression (*geneExp*) and mutation (*geneVar*).

w' represents the weight for genetic mutation. $*$ represents the vector dot product.

Self-attention:

$Attention(Q,K,V) = softmax(a(Q,K))V$

Q, *K* represent SMILES of compounds.
V represents weight parameters of gene weight layer.
$a(Q, K)$ is an alignment function which gives scores how well the inputs and the outputs match, and they normalized the scores by softmax function.

Training of parameters is performed using:
Loss function = mean-squared error
Optimizer = ADAM

References:
1. Kuenzi, B. M. *et al.* Predicting Drug Response and Synergy Using a Deep Learning Model of Human Cancer Cells. *Cancer Cell* **38**, 672-684.e6 (2020).
2. Jin, I. & Nam, H. HiDRA: Hierarchical Network for Drug Response Prediction with Attention. *J Chem Inf Model* **61**, 3858–3867 (2021).
3. Deng, L. *et al.* Pathway-Guided Deep Neural Network toward Interpretable and Predictive Modeling of Drug Sensitivity. *J Chem Inf Model* **60**, 4497–4505 (2020).
4. Manica, M. *et al.* Toward Explainable Anticancer Compound Sensitivity Prediction via Multimodal Attention-Based Convolutional Encoders. *Mol Pharm* **16**, 4797–4806 (2019).
5. Zhang, H., Chen, Y. & Li, F. Predicting Anticancer Drug Response With Deep Learning Constrained by Signaling Pathways. *Frontiers in Bioinformatics* **1**, (2021).
6. Koras, K., Kizling, E., Juraeva, D., Staub, E. & Szczurek, E. Interpretable deep recommender system model for prediction of kinase inhibitor efficacy across cancer cell lines. *Sci Rep* **11**, 15993 (2021).
7. Huang, X. *et al.* ParsVNN: parsimony visible neural networks for uncovering cancer-specific and drug-sensitive genes and pathways. *NAR Genom Bioinform* **3**, (2021).
8. Sakellaropoulos, T. *et al.* A Deep Learning Framework for Predicting Response to Therapy in Cancer. *Cell Rep* **29**, 3367-3373.e4 (2019).
9. Zuo, Z. *et al.* SWnet: a deep learning model for drug response prediction from cancer genomic signatures and compound chemical structures. *BMC Bioinformatics* **22**, 434 (2021).
